# The Outcomes of Organizational Cronyism: A Social Exchange Theory Perspective

**DOI:** 10.3389/fpsyg.2022.805262

**Published:** 2022-05-05

**Authors:** Shahab Ali, Farrukh Shahzad, Iftikhar Hussain, Pu Yongjian, Muhammad Mahroof Khan, Zafar Iqbal

**Affiliations:** ^1^School of Economics and Business Administration, Chongqing University, Chongqing, China; ^2^School of Economics and Management, Guangdong University of Petrochemical Technology, Maoming, China; ^3^Department of Public Administration, Faculty of Management Sciences, University of Kotli Azad Jammu & Kashmir, Kotli, Pakistan; ^4^Department of Economics, Faculty of Social Sciences & Humanities, University of Kotli Azad Jammu & Kashmir, Kotli, Pakistan; ^5^Department of Business Administration, Faculty of Management Sciences, University of Kotli Azad Jammu & Kashmir, Kotli, Pakistan

**Keywords:** cronyism, violation of psychological contract, organizational deviance, organizational cynicism, counter productive work behavior, social exchange theory

## Abstract

The current research examines the possible outcomes of cronyism like organizational deviance (OD), organizational cynicism (OCy), and counterproductive work behavior and also investigates the mediating variable violation of psychological contract (VPC) among cronyism and its possible outcomes. Many studies have investigated the presence of organizational cronyism (OC) at the workplace and its impacts on certain variables. However, the outcomes observed in this study, i.e., OD, OCy, and counter-productive work behavior were not empirically investigated previously as per researchers’ knowledge. The second gap this study fills is the mediating effect of VPC between the studied variables. Thirdly, the study was conducted in Azad Jammu and Kashmir, Pakistan, which is almost the first attempt to investigate this phenomenon in Azad Jammu and Kashmir. Data were collected from the employees working under different ministries of Azad Jammu and Kashmir, Pakistan like education, forest, sports, information, local government, finance, and tourism. The data from 350 employees were collected through convenience sampling. The data collection process was conducted at two distinct time lags. Results show that OC significantly and positively relates with OD, OCy, and counter-productive work behavior, whereas VPC mediates the relationship among OC and OD, OC, and counter-productive work behavior. Employees enjoying special favors from the leadership seem to be more dedicated toward the organization than the employees who do not have this favor, and the ultimate result is negative for the organization.

## Introduction

Organizational cronyism (OC) is the practice of conferring favors on coworkers, acquaintances, and those who have personal relationships with the leaders (Turhan, 2014). OC is like antimeritocracy behavior, which got a lot of attention by different researchers. Numerous researchers have found multiple precursors of OC like nepotism, favoritism, and particularism, which develop as a result of in-group prejudice and lead to OC (Khatri et al., 2006; Khatri, 2016). Likewise, paternalism fosters individual devotion toward OC. Finally, OC leads to a variety of attitudes and behaviors, including job dissatisfaction, organizational deviance (OD), counterproductive work behavior, ingratiation, OC, and low organizational commitment (Shaheen et al., 2019, 2021; Yu et al., 2021). Recently, many scholars recognized distinct types of OC, such as vertical and horizontal cronyism (Khatri et al., 2006). Favoring people at the same level, classification, or class, for example, friends, coworkers, social groups, class fellows, is horizontal form of cronyism, whereas providing undue benefits by the management to their close workers apart from their performance and favoring them by providing supportive environment and undue upgrades is vertical cronyism. According to Turhan (2014) workers who got improper perks, unjustifiable favors, and caring at others’ expenses are referred to as cronies. Although abundant evidence in the present literature that OC has significant human and organizational repercussions, few research studies are on attitudinal reactions of OC and how the concept of OC interprets into attitudinal behavioral outcomes.

Argyris (1960) and CLevinson (1962) were the first to discuss the contractual connections that exist between employees and employers in organizations, as well as the reciprocal commitments that exist between employees and employers. According to Schein (1978), employees have certain prior experiences and expectations that they utilize to generate present employer needs. However, expectations and wants are always changing and psychological contracts shift with time. Employers demand knowledge, skills, talents, dedication, and time from employees, whereas employees expect fairness, trust, support, recognition, appreciation, and monetary rewards from employers (Argyris, 1960). Violation of psychological contract (VPC) is significantly related to equity (Conway et al., 2014; Yang et al., 2020). According to Adams (1965), equity theory is applied when employees see inequality and injustice in efforts and results and then recognize that the organization does not have value for them. VPC arises by means of responding in a reasonable manner. A large body of study on organizational justice and favoritism shows that inequality and bias contribute to VPC. OD has received a great deal of consideration due to the serious outcomes (Samnani et al., 2014; Qiuyun et al., 2020; Mackey et al., 2021). Researchers have found many reasons for OD like abusive supervision (Tepper et al., 2009), less attention (Jones, 2009), and feeling of injustice (Hershcovis et al., 2007; Gong et al., 2021). Injustice and inequality, according to Jones (2009), are essential components in the development of aberrant workplace behavior. Current research contributes significantly to the existing literature by investigating OC, VPC, OD, organizational cynicism (OCy), CPWB, and association among them, and also identifying the gaps which are missing in literature. The objective of the present research focuses on investigating the attitudinal aspects of cronyism in governmental sectors of Azad Jammu and Kashmir, Pakistan in different ways that have not been studied yet. Firstly, to find out the relationship of OC on OD, OCy, and CPWB, and secondly the mediating effect of VPC among OC on OD, OCy, and CPWB.

Many studies have investigated the presence of OC at the workplace and its impacts on certain variables (Jawahar et al., 2021; Shah et al., 2021; Shaheen, 2021). However, the outcomes observed in this study, i.e., OD, OCy, and CPWB, were not empirically investigated previously as per researchers’ knowledge. The second gap this study fills is the mediating effect of VPC between the studied variables. Thirdly, the study was conducted in Azad Jammu and Kashmir, Pakistan, which is almost a first attempt to investigate this phenomenon in Azad Jammu and Kashmir. Therefore, this study was designed with the aim of measuring the impact of OC on OD, OCy, and CPWB. The study also examined the mediating effect of violation of psychological contract between the OC, OD, OCy, and counter-productive work behavior.

## Literature Review

### Organizational Cronyism and Organizational Deviance

Employee damaging and dishonest behavior endangers organizations and the well-being of its employees (Yen and Teng, 2013; Nauman et al., 2020). Unhelpful and harmful actions come at a financial, social, and psychological cost (Harvey et al., 2016). Absenteeism, stealing, fraud, abuse, stealing, vandalism, and sabotage are all behaviors identified by researchers. Retaliatory conduct, revenge, antisocial behavior, aggressiveness, and misbehavior are examples of undesirable behaviors (Penney et al., 2003; Tuna et al., 2016; de Walque, 2020). OD has grown in prominence among all other negative behaviors, which are defined as deliberate activity that harms the work-place and the well-being of the employees (Robinson and Bennett, 1995). According to Kickul (2001), similar practices occur in 95% of the companies. Robinson and Bennett (1995) categorize it as OD toward individuals/interpersonal and deviant workplace behavior toward organization. OD is defined as actions that contradict certain corporate principles and norms, such as theft, withdrawal attempts, absenteeism, tardiness, and sabotage, as well as stealing and abusing organizational property. According to Robinson and Bennett (1995), it is critical to categorize employee behavior in relation to their objectives to identify causes of deviation because interpersonal and OD are conceptualized differently. The role of organization cronyism as a predictor of aberrant workplace conduct has not been experimentally investigated. As a result, the researcher aims to work an optimistic link between OC and OD lace behavior using literature from injustice and social exchange theory. Some research, however, show that it is dependent on situational and contextual elements (Robinson and Bennett, 1995; Khan et al., 2013; Baharom et al., 2017; Azizi et al., 2021). The study finds inequality is a major cause of sabotage, and employees who face injustice engage in retaliatory measures to restore equity. The theory of social exchange Blau (1968) provides substantial theoretical evidence for the link between organizational favoritism and deviant workplace conduct. According to the reciprocity norm, when employees face injustice, they retaliate by engaging in destructive conduct and seek to restore equality by decreasing positive and increasing negative actions. As a result, from the above-stated literature, we can hypothesize that

*H1: There is a positive and significant relationship among organizational cronyism with organizational deviance.*

### Organizational Cronyism and Organizational Cynicism

Word Cynicism was first introduced by Kanter and Mirvis (1989) in the book “The Cynical Americans.” Employees who are cynical exhibit a lack of trust in both the organization and management. Employees of the organization feel that organization is not treating all the employees equally, where some employees are given special treatment while some employees are ignored. They had a sense of unfairness and thought that they had been used and treated unfairly by their organization. According to Kanter and Mirvis (1989), cynicism is defined according to sociological perspectives, “in cynicism, employees react unpleasantly with less confidence” (Polatcan and Titrek, 2014). According to Andersson (1996), it is a negative attitude toward the company or specific personnel. According to experts, there are two major elements that lead to the development of a cynical attitude, organizational, and interpersonal. Interpersonal factors can be marital status, educational level, salary, gender, and experience. Organizational issues include VPC, organizational fairness, and role conflict. According to the definition by Dean et al. (1998) cynicism as well as its aspects and cynicism as negative feelings toward organizations are classified into three aspects: (1) employee feels that the organization appears to be dishonest; (2) unfavorable sentiments against the organization; and (3) proclivity to exhibit bad conduct toward the organization. Furthermore, academics define several forms of cynicism, including civil servant cynicism, societal cynicism, OCy, and job cynicism. When people believe they are not being treated fairly and that their organizations are failing to meet their stated duties, emotions of distrust and dissatisfaction grow (Adams, 1965). Unfairness and injustice are key characteristics of cronyism. Furthermore, the norm of negative reciprocity depicts that when workers are treated poorly by their boss and organization as a result, they respond with bad attitudes, i.e., cynicism (Gouldner, 1960; Mughal, 2020; Mousa et al., 2021). Thus, we can hypothesize based on the above-mentioned literature.

*H2: There is a positive relation between Organizational Cronyism and Organizational Cynicism.*

### Organizational Cronyism and CPWB

Counter productive work behavior is described as purposeful carelessness that disturbs the working of the organization. According to Hirschman (1970), the CPWB has serious human and organizational outcomes, and it is more harmful than other damaging behaviors, i.e., voice (to stand up for legal rights) and commitment (pretending loyalty for the organization). When employees face inequity, the majority of them are motivated to leave the workplace, both psychologically and physically, whereas those who remain in the organization despite the danger of bias engage in irresponsible behavior (Meisler and Vigoda-Gadot, 2014; Bilal et al., 2020; De Clercq et al., 2021). Workers remain physically present but mentally absent in this particular situation, and most of them waste the organization’s time by purposely working slowly, postponing tasks for no reason, and ultimately showing no output from their side toward the organizational development. Employees get frustrated, less dedicated, and less motivated when they believe they are not treated equally despite possessing the required skills and abilities. Employees start irresponsible activities when they feel a sense of injustice at the workplace. Negative acts are repaid with negative behaviors in response (Blau, 1968). As a result, employees who have experienced injustice act negatively for organizations and involve in the CPWB, which is very harmful to organizations. So we can hypothesize,

*H3: There is positive and significant relation among organizational cronyism and counter productive work behavior.*

### Violation of Psychological Contract and Organizational Deviance

Numerous academics have found unfavorable workplace behaviors such as abusive supervision, despotic leadership, nepotism, favoritism, drug and alcohol usage, and fraud, and such activities have a negative influence on 95% of the organizations (Kickul, 2001). Negative behaviors include workplace incivility, unpleasant and disrespectful conduct, antisocial and organizational misbehavior according to Sayers et al. (2011), and such actions affect the business as well as the people associated with it, such as stakeholders’ workers and consumers. OD has received a lot of consideration by the researchers due to serious consequences (Samnani et al., 2014), and a number of OD antecedents have been noticed by researchers, i.e., abusive supervision (Tepper et al., 2009) and perceived injustice (Cohen-Charash and Mueller, 2007; Hershcovis et al., 2007). Injustice and inequality, according to Jones (2009), are essential components in the development of an aberrant workplace behavior.

Social exchange theory and the norm of negative reciprocity give theoretical basis for establishing a positive link between VPC and OD (Berry et al., 2007). When employees perceive that their commitments have not been recognized, they respond by reducing constructive behaviors and increasing damaging behaviors (Uhl-Bien and Maslyn, 2003; Balogun et al., 2018; Shaffakat et al., 2021). Furthermore, unfulfilled commitments by the organization results in rage, dissatisfaction, absenteeism, job neglect, distrust, destruction, poor OCB, and high OD (Robinson and Bennett, 1995; Jiang et al., 2017; Asante et al., 2021). As a result, workers attempt to reestablish equality by engaging in bad conduct in reaction to their unfulfilled psychological contract. So, from the above literature, we can hypothesize that

*H4: Violation of psychological contract mediates the relationship between organizational cronyism and organizational deviance.*

### Violation of Psychological Contract and Organizational Cynicism

Employee expectations from their employer in terms of better working conditions, compensation, advancement, and equitable benefits in return of their services are referred to as psychological contracts (Robinson et al., 1994; Rousseau, 1998; Hui et al., 2004). However, VPC occurs when an employee encounters inequality and believes that he is not getting as much appreciation/reward in return of his/her contribution. Employees perceive it as unmet promises made on the behalf of the organization (Morrison and Robinson, 1997). Social exchange theory by Blau (1968) also contributes to a better understanding of the psychological contract among workers and management. OCy is referred to as a wider object and studies associate cynicism with a variety of objects (Bateman and Organ, 1983; Andersson, 1996). The major consequences of VPC are employee perceptions of the organization’s lack of integrity and cynical conduct (Sarikaya and Bayrak Kök, 2017; Li and Chen, 2018; Lapointe et al., 2020). Johnson and O’Leary-Kelly (2003) investigated the role of OCy as a mediator in the relationship among CPWB and VPC. Researchers discovered that cynicism partially mediates the link among VPC and OC and work satisfaction, but completely mediates the relationship among emotional exhaustion and VPC by concentrating on banking sector employees. Furthermore, VPC is related to worse organizational performance and higher absenteeism. So we can hypothesize that

*H5: Violation of psychological contract mediates the relationship between organizational cronyism and organizational cynicism.*

### Violation of Psychological Contract and CPWB

A psychological contract is described as mutual expectations shared between both parties, such as an employee and an employer, or an employee and an organization. It is a written or unwritten trade agreement among both the parties (Argyris, 1960). Once an employee feels that there is a contradiction between psychological contracts and the organization is unable to fulfill the promises, this leads them toward VPC (Shaheen et al., 2017; Griep and Vantilborgh, 2018; Griep et al., 2020; Piccoli et al., 2021). Social exchange theory, control theory, and cognitive dissonance theory are the most popular theories to know about employees–employers relation, he relationship among employees and organizations, and to understand the actual cause of VPC (Zagenczyk et al., 2015). Researchers investigated if VPC has an unfavorable influence on employee attitudes and actions. According to Turnley and Feldman (1999), VPC is favorably related with voice, neglect, and departure while being adversely associated with loyalty. Active answers are beneficial to both individuals and organizations, but passive replies are detrimental to both the organization and employee’s well-being. As a result, passive behaviors, i.e., negligence and intention to leave the organization are regarded as critical for the organization and the workers as well. As a result, this research aims to investigate the impact of VPC on CPWB.

*H6: Violation of psychological contract mediates the relationship between organizational cronyism and counter-productive work behavior.*

**FIGURE 1:**
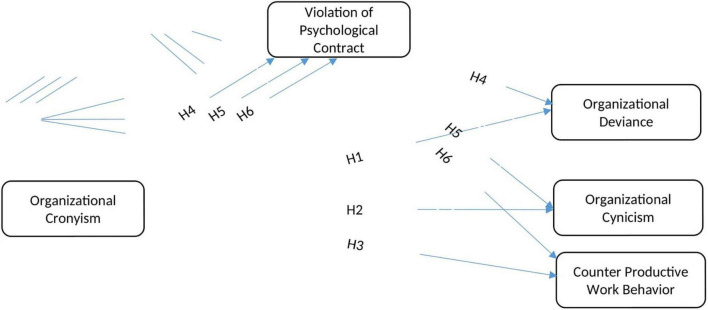
Research model.

To check whether VPC mediates the relationship among OC and OD, OCy, and CPWB, the equational form of variables is carried out with the help of the steps proposed by Wu and Zumbo (2008), and the development of hypothesis is completed on the basis of extensive literature review (Figure 1).

1.1:


(9.1.a) Patha:VPC=f(OC)VPC=∝+β1(OC)+e------

(9.1.b) Pathb:OD=f(VPC)OD=∝+β1(VPC)+e------

Pathc:OD=f(OC,VPC)OD=∝+β1(OC)*β2(VPC)

(9.1.c) +e-----

1.2:


(9.2.a) Patha:VPC=f(OC)VPC=∝+β1(OC)+e-------

(9.2.b) Pathb:OCy=f(VPC)OCy=∝+β1(VPC)+e-----

Pathc:OCy=f(OC,VPC)CY=∝+β1(OC)*β2(VPC)

(9.2.c) +e-------

1.3:


(9.3.a) Patha:VPC=f(OC)VPC=∝+β1(OC)+e------

(9.3.b) Pathb:CPWB=f(OC)CPWB=∝+β1(OC)+e-----

Pathc:CPWB=f(OC,VPC)CPWB=∝+β1(OC)*

(9.3.c) β2(VPC)+e-------

## Research Methodology

### Sample and Data Collection

This study’s population consists of personnel from several ministerial offices of Muzaffarabad capital of Azad Jammu and Kashmir ministerial offices, including education, forest, sports, information, local government, finance, and tourism (Fu et al., 2021). Researchers visited the concerned offices and briefly presented the topic of study. Researchers contacted the HODs of HRM and conveyed the goal of research to them and ensured them that collected data will not be misused and will be published for improvement purposes of the concerned departments. The data was collected in 2-time waves because of its authenticity; an introductory letter was placed in front of the questionnaire which had detailed information about the purpose of research. We had gathered the data from 350 employees, of which 20% were women and 80% were men, 32% were between the ages of 25 and 35, 40% between the ages of 35 and 45, and 28% between the ages of 45 and 60, 45% had a bachelor’s degree and 55% had a master’s degree, 30% had less than 3 years of experience, 25% had 3 to 5 years of experience, and 45% had more than 5 years of experience.

#### Measure

To assess the focus constructs, we employed previously validated questions. Five-point Likert scale is used to test the measure and rated as (1). Strongly disagree, (2). Disagree, (3). Neutral, (4). Agree, and (5). Strongly agree.

#### Organizational Cronyism

Turhan (2014) created a 15-item scale for measuring OC. “Our boss treats employees with whom he has a closer personal connection with greater tolerance,” “In our institution, workers are paid based on their performance rather than their personal relationships with the management,” or “When settling disagreements, our manager protects employees with whom he has a deeper personal connection.”

#### Violation of Psychological Contract

Robinson and Bennett (1995) designed five items scale to check VPC. Restubog et al. (2007) and Sayers et al. (2011) used this measure in their researches “Almost all of my employer’s promises made to me during recruiting have been maintained so far,” “I believe that my employer has come through in keeping the promises made to me when I was employed,” and “So far, my employer has done a great job of delivering its promises to me.”

#### Organizational Deviance

Nineteen item scale is used by Bennett and Robinson (2000) to access the OD. Other researchers also used this measure in their research, i.e., (O’Neill and Hastings, 2011; Yen and Teng, 2013). Some of the questions include “Made fun of someone at work,” “Said something nasty to someone at work,” and “Made an ethnic, religious, or racial comment at work” are some sample scale items used in this study to measure OD.

#### Organizational Cynicism

Dean et al. (1998) used a five item scale to measure OCy. Items include “I believe my organization says one thing and does another,” “Policies, aims, and practices of my organization appears to have little in common,” “When my organization promises it will accomplish something, I’m not sure if that will actually happen.”

#### Counter Productive Work Behavior

The scale established by Vigoda-Gadot and Meisler (2010) was used to assess employees’ CPWB. The four-item measure aided in understanding workplace employee carelessness. “Sometimes I put off essential assignments for an indefinite length of time,” “Sometimes I don’t perform all of my obligations at work,” and “This institution doesn’t care much about people like me, therefore I’m not willing to put in additional effort for it” are some sample scale items used to measure CPWB.

## Results

Analysis of moment structures (AMOS) is used for statistical analysis, and it is most commonly used in structural equation modeling. Reasons to use analysis of moment structure are as follows: (1) To carry out the statistical analysis flawlessly, accurately, and competently; and (2) AMOS based on covariance SEM (Hair et al., 1998). It is also used to test the theories, as we are testing theory in this study, we opted to employ this particular statistical analysis. Furthermore, we have to perform CFA in this research so AMOS is reasonable for such working as suggested by previous researchers (Hair et al., 2006; Abbas et al., 2019). As a result, we feel AMOS is an ideal statistical instrument for testing our suggested model. The test for Mediation Analysis was performed using Structural Path in AMOS.

### Reliability, Validity, and Correlation Analysis

Reliability was measured using values of composite reliability (CR). Fornell and Larcker (1981) recommended that for data reliability, CR values should be 0.70 or higher. The values of CR for all constructs in the current study are well above the set criteria as shown in Table 1. Confirmatory factor analysis (CFA) was conducted to examine validities, such as discriminant and convergent validity. As suggested by Bagozzi and Yi (1988), convergent validity can be measured with the values of CR, items’ standardized factor loading, and average variance extracted (AVE). CR values greater than 0.70, items’ standardized factor loading, and AVE values greater than 0.50 show excellent convergent validity (Bagozzi and Yi, 1988). Results shown in Table 1 fulfill Bagozzi and Yi’s (1988) conditions for convergent validity. According to Fornell and Larcker (1981), discriminant validity can be established through the greater values for square root of AVE than the construct’s correlation values and higher AVE values than the maximum share variance (MSV) values. Results shown in Table 1 fulfill the Fornell and Larcker’s (1981) criteria.

**TABLE 1:** Reliability, validity, and correlation analysis.

Variables	CR	AVE	MSV	1	2	3	4	5	
OC	0.89	0.58	0.23	−0.049	**0.775**				
VPC	0.85	0.55	0.37	−0.009	0.319**	**0.744**			
OD	0.86	0.56	0.23	−0.051	0.516**	0.605**	**0.723**		
OCY	0.92	0.62	0.37	0.197**	0.333**	0.339**	0.488**	**0.774**	
CPWB	0.91	0.61	0.24	−0.040	0.584**	0.490**	0.597**	0.363**	**0.738**

The symbol ** means the significance level is 0.05. Bold indicates they are lying in an acceptable range.

Table 1 shows that, OC is positively and significantly related to the VPC (*r* = 0.319, *p* < 0.05), OD (*r* = 0.516, *p* < 0.05), OCy (*r* = 0.333, *p* < 0.05), and CPWB (*r* = 0.584, *p* < 0.05), whereas VPC is significantly related to OD (*r* = 0.605, *p* < 0.05), OCy (*r* = 0.339, *p* < 0.05), and CPWB (*r* = 0.490, *p* < 0.05).

### Confirmatory Factor Analysis (CFA)

AMOS is used to test the hypothesis, and the measurement model was evaluated using confirmatory factor analysis (CFA). Model fitness is measured using the “IFI,” “TLI,” “comparative fit index (CFI),” and “root mean square error of approximation (RMSEA).” There are five theoretical variables in the proposed model: independent variable (OC), mediating variable (VPC), and three dependent variables (OD, OCy, and CPWB). Table 2 shows that the model fitness is low initially since all “IFI,” “TLI,” “CFI,” and “root mean square error of approximation (RMSEA)” values are not much reliable. So for suitable model fitness many changes were performed. We achieved good model fitness after a number of changes, as evidenced by “IFI = 0.95,” “TLI = 0.92,” “CFI = 0.95,” and “RMSEA = 0.05.”

**TABLE 2:** Model fit summary.

	Chi- Square	Df	CMIN/DF	IFI	TLI	CFI	RMSEA
Early model	3153.613	825	3.822	0.65	0.64	0.65	0.11
Modified model	1625.23	618	2.62	0.95	0.92	0.95	0.05

### Hypothesis Verification

The results of Hypothesis 4, 5, and 6 supported a mediating function of the VPC in the link between OC and OD, OCy, and CPWB, as seen in Table 3. Link between OC and the occurrence of a VPC is significant (β = 0.19, *p* < 0.05), (β = 0.08, *p* < 0.05), and (β = 0.09, *p* < 0.05), but it has been decreased in the presence, demonstrating partial mediation. As a result, VPC mediates the relation between OC and OD, OCy, and CPWB to some extent as indicated in Table 4.

**TABLE 3:** Direct paths.

Structural path	Path coefficient	SE	*P*-value
OC→OD	0.52	0.02	***
OC→OCY	0.33	0.04	***
OC→CPWB	0.78	0.03	***

The symbol *** means the significance level is 0.10.

**TABLE 4:** Mediation effects.

Structural path	Indirect effect	BC (95% CI)
OC→VPC→OD	0.19	(0.12, 0.25)
OC→VPC→OCy	0.08	(0.04, 0.12)
OC→VPC→CPWB	0.09	(0.05, 0.13)

## Conclusion

Organizational cronyism has recently received a lot of attention from academic scholars and professionals owing to its negative repercussions. The authors explore OC as a precursor to OD, OCy, and CPWB by adding to the current body of research. The current study additionally looked at VPC as a mediator among the OC and OD, OCy, and CPWB.

All the research hypotheses received strong empirical evidence. Our data show that OC is connected to VPC and OD, OCy, and CPWB, and that VPC also mediates the association between cronyism and OD, OCy, and CPWB. The results of the study support the idea that non-crony workers respond to OC through OD, OCy and CPWB. These conclusions are like prior studies that asserted that employees are more prone to engage in deviance under situations that encourage OC (Kelloway et al., 2010).

Employees who are passionate about the organization and have worked hard to fulfill organizational obligations confront considerable challenges as a result of OC, which denies them promotions, polite treatment, and progression possibilities. These situations give rise to perceptions of injustice, which contribute to the VPC sensation. Employees frequently participate in OD, OCy, and CPWB in reaction to VPC to restore fairness in their organizational relationships (Adams, 1965). OD, OCy, and CPWB can have an effect on organizational performance by reducing the morale of the employees (Shaw et al., 2005; Quratulain and Khan, 2015). Cronyism is so prevalent in government organizations with a belief that violations of the psychological contract (VPC) take place frequently, and as a result, employees engage in OD, OCy, and CPWB. Due to a lack of accountability, incompetence, and dishonesty are widespread in many governmental institutions in underdeveloped nations. A unified voice throughout the globe asserts that equal treatment should be given to all the employees in government institutes, although research has been limited to multinational corporations and industrialized nations. Current research revealed the prevalence of OC in the organizations working under the government of Azad Jammu and Kashmir, and also demonstrated how it can be a reason for OD for many employees.

The research analysis gives a genuine image of Azad Jammu and Kashmir’s public sector institutions. Cronies perform better and shine at work more than non-cronies because they have strong ties with the boss and can gain undue favors. Secondly, another factor that is involved is the non-merit selection of the employees who do not have enough skills and abilities to perform the required takes in the organization, and they enjoy the financial benefits of the organization as well as they get relaxation in required duties like assignments, working hours, commitments, etc. We have found that OC is a major contributor to the emergence of these harmful workplace practices. Current research also shows a clear image of AJ&K public sector organizations, where favoritism and nepotism take precedence over real knowledge, skills, and competence, and employees strive to preserve harmonious relationships with the boss rather than focusing on their jobs.

### Managerial and Theoretical Implications of Research

There are some theoretical and managerial implications to the current research work. Researchers attempted to add research on OC and its possible outcomes. The authors used the VPC to test the influence of OC on workers’ reactions. An empirical study of OC and its outcomes also filled a vacuum in the literature identified by Khatri and Varma (2019). According to the current study, workers who are closed to the managers can get benefits as compared to the employees who do not have this situation, but in long-term it is very harmful for the organization. As a result, a crony exhibits additional good and fewer bad conduct. According to the study’s findings, OC promotes workers to engage in ingratiatory methods to gain favor and confidence, and to preserve cordial connections with an immediate boss for long-term. By using such practices, cronies gain advantages over non-cronies. Allowing fairness in recruitment and selection on merit to grow in companies is one of the most effective methods to put a halt to such behaviors. Managers, particularly in government organizations, must understand that due to education and experience most of the employees are well aware of their rights and when VPC occurs due to OC employees might move toward service tribunals for justice, which will ultimately damage the goodwill of the organization. Additionally, another appropriate way is to inspire top leadership in public companies to promote merit-based judgments instead of influential decisions.

### Limitations and Prospects for Future Research

The results of this research have consequences for government sector organizations, some of which are discussed below. Researchers have demonstrated that the OC in the workplace drives people to engage in OD, OCy, and CPWB. All these outcomes of cronyism are very bad for organizational productivity and public service delivery. Establishing merit-based procedures in public institutions is one method to decrease OD, OCy, and CPWB. Managers in the public sectors must be trained and must have up-to-date knowledge and skills to deal with subordinates and must show a sense of equality among the employees. Merit-based practices should be encouraged. Public sector firms might also try to improve their employees’ views of fairness. It is possible to do this by implementing fair processes for resource distribution, as individuals are prepared to tolerate bad consequences if they believe that there has been a fair distribution of organizational resources without any discrimination (Folger et al., 1979). Managers’ conduct can also help to decrease perceptions of corporate cronyism (Hoy and Tarter, 2004).

A more egalitarian, empathetic, and dedicated toward boss may be seen as loyal. Unbiased conduct does not just refer to incentives or promotions, but also refers to issues such as providing a better working environment, limited involvement in policymaking/decision making, public acknowledgment, and so on. Top-level government. officers should pay attention to such elements to improve their employees’ views of fairness. Our research findings and their consequences have some limitations as well. First, researchers just looked at one result of OC, and it is possible that there maybe are other behavioral factors involved which can agree or disagree with the findings. OCB, ingratiation, and intention to quit can be some variables that can be studied with OC. Second, a bigger sample size research in the future can give evidence for the generalization of current results. Third, the researcher has taken population from Azad Jammu and Kashmir, Pakistan which is part of an underdeveloped country; further research can be made by targeting population from any developed country like United States, United Kingdom, or Europe.

## Data Availability Statement

The raw data supporting the conclusions of this article will be made available by the authors, without undue reservation.

## Author Contributions

SA completed introduction, literature, methodology, discussion, conclusion, and wrote and edited original draft. IH conceptualized the idea, writing original draft, and edited the original manuscript. PY supervised this project, funding, edited original draft, and data collection. MK contributed in methodology and analysis. ZI conceptualization and methodology. FS was involved in writing and editing the manuscript as well as in analyzing results, and funding acquisition was his major contribution. All authors have read and agreed to the published version of the manuscript.

## Conflict of Interest

The authors declare that the research was conducted in the absence of any commercial or financial relationships that could be construed as a potential conflict of interest.

## Publisher’s Note

All claims expressed in this article are solely those of the authors and do not necessarily represent those of their affiliated organizations, or those of the publisher, the editors and the reviewers. Any product that may be evaluated in this article, or claim that may be made by its manufacturer, is not guaranteed or endorsed by the publisher.
